# Exploring Electrochemical Direct Writing Machining of Patterned Microstructures on Zr702 with Polyacrylamide Polymer Electrolyte

**DOI:** 10.3390/mi15091074

**Published:** 2024-08-26

**Authors:** Junfeng He, Wenjie Chen, Junjie Wang, Ming Wu, Li Zhou, Ri Chen, Huazhuo Liang

**Affiliations:** 1 School of Mechatronic Engineering, Guangdong Polytechnic Normal University, Guangzhou 510665, China; hejunfeng@gpnu.edu.cn (J.H.);; 2 School of Mechanical and Automotive Engineering, South China University of Technology, Guangzhou 510641, China; 3 Department of Architectural Engineering, Guangzhou Panyu Polytechnic, Guangzhou 511487, China; 4 Department of Computer Science, Leuven AI, KU Leuven, 3001 Leuven, Belgium; 5 Department of Mechanical Engineering, KU Leuven, 3001 Leuven, Belgium; 6 Interdisciplinary Research Institute, Guangdong Polytechnic Normal University, Guangzhou 510665, China; 7 Research Institute of Heyuan, Guangdong Polytechnic Normal University, Heyuan 517500, China

**Keywords:** polyacrylamide polymer electrolyte, direct writing, patterned microstructure, electrochemical machining

## Abstract

Zirconium alloys possess excellent wear resistance, which ensures the durability and longevity of the components, making them widely used in medical and other fields. To enhance the functionality of these materials, it is often necessary to fabricate functional microstructures on their surfaces. Electrochemical machining (ECM) techniques demonstrate excellent machining performance for these metals, particularly in the processing of microstructures on complex curved surfaces. However, ECM often faces challenges due to the fluid nature of the electrolyte, resulting in low machining accuracy and localization. This paper proposes a novel method for fabricating complex patterned microstructures using a maskless electrochemical direct writing technique with a polyacrylamide (PAM) polymer electrolyte. By leveraging the non-Newtonian properties of PAM, this method effectively confines the electrolyte to specific areas, thus addressing the issue of poor localization in traditional ECM and reducing stray corrosion. To elucidate the electrochemical removal mechanism of Zr702 in the presence of PAM, polarization curves, viscosity characteristics, and current efficiency parameters were analyzed. Additionally, an experimental study was conducted using a custom-designed nozzle structure. The results showed that the PAM electrolyte could effectively reduce the EF, positively impacting machining accuracy and localization. By controlling the nozzle’s motion trajectory, complex microstructures were successfully fabricated through direct writing, demonstrating promising application prospects.

## 1. Introduction

Zirconium (Zr) is known for its outstanding passivating characteristics. The oxide films that develop on passivated metals offer resistance to corrosion from strong acids and alkalis, high-temperature water, and liquid metals [1,2]. While zirconium alloys may not possess the hardness of titanium alloy, they excel in wear resistance, thereby ensuring prolonged durability of the components. For example, they are highly regarded in the medical sector for their exceptional corrosion resistance, wear resistance, and biocompatibility. Specifically, they are used in the fabrication of artificial bones, medical instruments, dental restorative materials, and various other medical devices. Currently, metal surface microstructures are frequently utilized to enhance the surface properties of metals and improve material applicability. However, due to the physical properties of Zr, microstructural processing of Zr surfaces remains challenging in the industry, particularly for complex patterned functional microstructures [3].

At present, the most commonly used microstructure processing technologies include mechanical processing and nontraditional manufacturing. In their study on the mechanical processing of microstructures on Zr alloys, Wang et al. [4] demonstrated that micro-pillar arrays manufactured by micro-milling on the surface of a Zr alloy exhibited good structural stability and dimensional accuracy, which contributed to improved corrosion resistance. However, Hood et al. [5] observed significant heat generation during the milling of a Zr alloy, making the workpiece and chips susceptible to oxidation. This resulted in significant residual stress on the machined surface, impacting the machining quality. In another study, Chen et al. [6] conducted drilling experiments using a 2 mm solid carbide drill on a new β dental Ti–Zr–Nb alloy. They found that the alloy’s high strength and hardness caused significant resistance to plastic deformation and severe cold hardening, necessitating a substantial drilling force. Furthermore, the metal’s low thermal conductivity led to heat accumulation at the cutting edges and chips during drilling, resulting in severe tool adhesion, which could accelerate tool wear and reduce tool life. Zr alloys possess robust physical properties that often pose challenges in traditional microstructure processing methods, resulting in high cutting forces and temperatures. These challenges frequently lead to substantial tool wear, a primary factor contributing to issues such as burrs, built-up edges, surface roughness, and subsurface damage [7].

Nontraditional manufacturing processes offer a more promising avenue for microfabrication as they are not limited by the hardness and strength of metals. Examples of such processes include electrical discharge machining (EDM), laser machining (LM), and electrochemical machining (ECM). EDM is a machining technology that uses electrical and thermal energy to process materials by melting and eroding them at high temperatures. Mu et al. [8] introduced an innovative approach to EDM called intelligent EDM, which aims to address issues related to microstructure machining rates and stability. The developed intelligent EDM demonstrated excellent machining capabilities. In their study, Kumar et al. [9] utilized Cu, CuW, and graphite tool electrodes to perform EDM on Zircaloy-2. They analyzed various machining results such as tool wear rate, surface roughness, and material removal rate. They found that the high melting point and poor thermal conductivity of Zircaloy-2 necessitated a high energy input for the discharge process. One of the challenges in EDM is significant tool electrode wear and the formation of recast layers, especially when machining closed microstructures, which can lead to a decrease in the quality of the microstructures [10,11]. LM, known for its lack of material selectivity and high processing efficiency, is a crucial method for processing microstructures. Yue et al. [12] introduced selective laser melting to manufacture commercially pure Zr components with excellent strength and plasticity, enabling the direct writing processing of patterned microstructures. Li et al. [13] employed femtosecond laser ablation to create microgrooves on zirconia oxide surfaces, resulting in a stable tetragonal crystal structure with a relatively intact morphology. However, laser processing often leads to the formation of a thermal affected zone, which can result in a rougher surface finish. Furthermore, an increase in processing depth is associated with a notable decrease in cutting speed [14].

ECM is a non-contact processing method in which materials dissolve via redox reactions, with no physical contact between the workpiece and the tool. This process avoids tool adhesion and residue formation [15,16]. Unlike EDM and laser processing, ECM tools do not result in any loss and do not produce thermal affected zones [17]. This method is particularly well-suited for machining high-strength and high-hardness metallic materials [18]. Chen et al. [19] proposed the use of jet electrolysis direct writing to fabricate microchannels. In this method, an insulated mask with a row of micro through-holes was integrated to a metallic nozzle; the electrolyte ejected from the nozzle then reached the workpiece through micro through-holes, and subsequently a micro-channel array could be generated from dots to lines by controlling the movement of the workpiece. However, according to Faraday’s law, the fluidity of the electrolyte poses a challenge in precisely controlling dimensional accuracy during processing, resulting in poor localization and significant deviations between actual and intended shapes [20]. To ensure the shape accuracy of functional microstructures, masks are often used to restrict the flow of the electrolyte [21,22]. However, for complex surface microstructures, the production of masks can be difficult, thereby limiting the widespread application of electrochemical machining.

Considering these challenges, this paper presents a novel method for manufacturing patterned microstructures using a maskless electrochemical direct writing (ECDW) technique with a polyacrylamide (PAM) polymer electrolyte. By leveraging the unique properties of non-Newtonian fluids, this approach effectively confines the electrolyte to specific areas without requiring a patterning mask. This method not only addresses the issue of stray corrosion inherently found in traditional ECM but also allows for the processing of patterned microstructures on complex surfaces using a three-dimensional motion platform. Through the use of a high-pressure pump, the polymer is extruded in accordance with the desired microstructure pattern, enabling the direct writing of microstructures onto the workpiece surface by controlling the current density and motion trajectory. This methodology facilitates the precise, efficient, and controllable fabrication of intricate patterned microstructures.

## 2. Material and Methods

### 2.1. Experimental Materials

PAM (Xinbang Environmental Protection Technology Co., Ltd. Yiwu, China) is a linear polymer widely used among water-soluble polymers, with the chemical formula ${\left(C_{3}H_{5}\mathrm{NO}\right)}_{n}$ and molecular weights ranging from 5 to 18 million. Generally, PAM products with a higher molecular weight have a higher viscosity. PAM is a colorless, odorless crystalline solid containing amide groups ($-{\mathrm{CONH}}_{2}$) in its basic structure. Its solubility is primarily attributed to these amide groups, which can form hydrogen bonds with water molecules, resulting in stable aqueous solutions. Consequently, PAM exhibits excellent solubility and surface activity. Its aqueous solutions are nearly transparent viscous liquids, exhibiting non-Newtonian flow behavior under stress. Furthermore, PAM demonstrates good thermal and chemical stability, remaining stable and resistant to degradation under ambient conditions. It has a density of 1.302 g/cm^3^ at 23 °C, with a glass transition temperature of 153 °C and a softening temperature of 210 °C.

**Figure 1:**
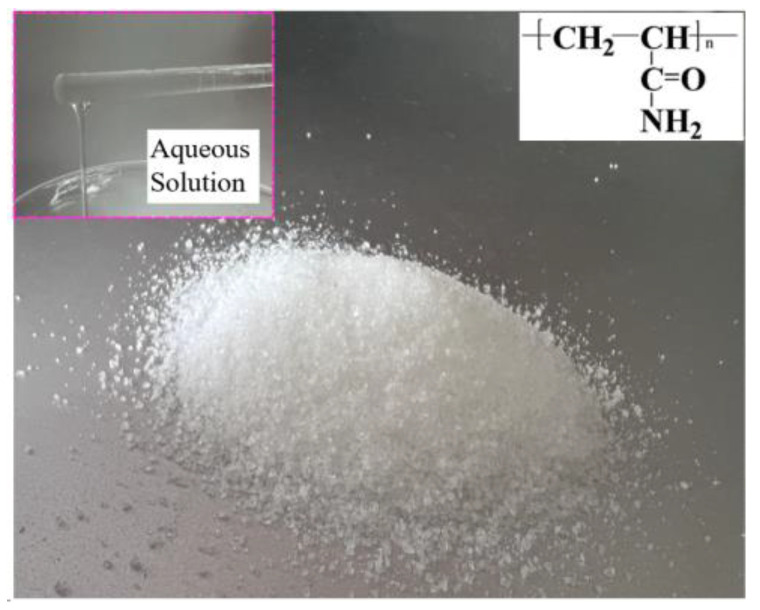
PAM and its molecular formula.

Due to its unique polar functional groups, PAM serves as an effective thickener, paper strengthening agent, and drag reduction agent in liquids. It can also serve as an adsorption bridge, causing suspended particles to aggregate and precipitate, thereby purifying wastewater. Commonly used in water purification, especially when combined with inorganic coagulants, it exhibits optimal performance in water treatment, making it an environmentally friendly polymer. In this study, non-ionic PAM was selected as the electrolyte to investigate the electrochemical properties of PAM (Figure 1).

PAM is typically synthesized through the hydration of acrylonitrile to produce the monomer acrylamide in the presence of a Cu catalyst. PAM is further polymerized under the action of a $K_{2}S_{2}O_{8}$ initiator. The redox initiation system plays a crucial role in the polymerization of acrylamide and is widely used in various applications. It can be categorized into four main types: persulfates, organic peroxides, multi-electron transfer redox systems, and non-peroxide systems. Additionally, acrylamide polymerization can also be initiated through methods such as radiation polymerization, thermal initiation, photo-initiation, precipitation polymerization, and micellar polymerization. The reactions can be represented by Equations (1) and (2):

(1) $${\mathrm{CH}}_{2}={\;\mathrm{CHCN}+H}_{2}O\overset{Cu}{\to }{\mathrm{CH}}_{2}={\mathrm{CHCONH}}_{2}$$

(2) $$\begin{array}{l} n{\mathrm{CH}}_{2}{=\mathrm{CHCONH}}_{2}\overset{K_{2}S_{2}O_{8}}{\to }-{\left[{\mathrm{CH}}_{2}\mathrm{CH}\right]}_{n}-\\ \;\;\;\;\;\;\;\;\;\;\;\;\;\;\;|\\ {\;\;\;\;\;\;\;\;\;\;\;\;\;\;\mathrm{CONH}}_{2} \end{array}$$

In aqueous solutions, polyacrylamide undergoes hydrolysis of the amide groups, resulting in the formation of polymers containing carboxyl groups. This reaction can be represented as Equation (3):

(3) $$\begin{array}{l} {\left[{\mathrm{CH}}_{2}{=\;\mathrm{CHCONH}}_{2}\right]}_{n}{+H}_{2}O\overset{H^{+}}{\underset{or\;OH^{-}}{\to }}{\left[-{\mathrm{CH}}_{2}-\;\mathrm{CH}-{\mathrm{CH}}_{2}-\;\mathrm{CH}-\right]}_{m}{+\mathrm{NH}}_{3}↑\\ \;\;\;\;\;\;\;\;\;\;\;\;\;\;\;\;\;\;\;\;|\;\;\;\;\;|\\ {\;\;\;\;\;\;\;\;\;\;\;\;\;\;\;\;\;\;\;\;\;\;\mathrm{CONH}}_{2}\;\;\;\;\;\;\;\;\;\;\mathrm{COOH} \end{array}$$

In aqueous solutions, −COOH  (carboxyl) can lose a proton ($H^{+}$) to form ${\mathrm{COO}}^{-}$ (carboxylate) ions. This deprotonation process involves the acceptance of the proton from the carboxyl group by water molecules in the solution, resulting in the formation of carboxylate ions.

(4) $$\begin{array}{l} \left[-{\mathrm{CH}}_{2}-\;\mathrm{CH}-{\mathrm{CH}}_{2}-\mathrm{CH}-\right]\to \left[-{\mathrm{CH}}_{2}-\;\mathrm{CH}-{\mathrm{CH}}_{2}-\;\mathrm{CH}-\right]\;+H^{+}\\ \;\;\;\;\;\;\;\;\;\;\;\;\;\;\;|\;\;\;\;\;|\;\;\;\;\;\;\;\;\;|\;\;\;\;\;|\\ {\;\;\;\;\mathrm{CONH}}_{2}{\;\;\;\;\;\;\;\;\;\mathrm{COOH}\;\;\;\;\;\;\;\mathrm{CONH}}_{2}{\;\;\;\;\;\;\;\;\;\;\mathrm{COO}}^{-} \end{array}$$

In the anode reaction, zirconium can ionize in water, resulting in the production of Zr^4+^ ions that enter the electrolyte, as shown in Equation (5).

(5) $$\mathrm{Zr}\to {\mathrm{Zr}}^{4+}{+4e}^{-}$$

The Zr^4+^ ions can further react with the carboxyl groups in the carboxylate ions, forming coordination bonds between the carboxyl groups and the metal ions to create stable complexes. The reaction is as shown in Equation (6).

(6) $$\begin{array}{l} {\left[-{\mathrm{CH}}_{2}-\;\mathrm{CH}-\right]}_{4}+{\mathrm{Zr}}^{4+}\to \left[-{\mathrm{CH}}_{2}-\;\mathrm{CH}-\right]\\ \;\;\;\;\;|\;\;\;\;\;\;\;\;\;\;\;\;|\;\;\;\;\;\;\;\;\;\;\;\;\\ {\;\;\;\;\;\mathrm{COO}}^{-}\;\;\;\mathrm{COOZr}\; \end{array}$$

The cathode reaction is the same as for most metal electrolysis reactions: $H^{+}$ is reduced to $H_{2}$. The reaction is as shown in Equation (7).

(7) $${2H}^{+}{+2e}^{-}{=H}_{2}↑$$

**Figure 2:**
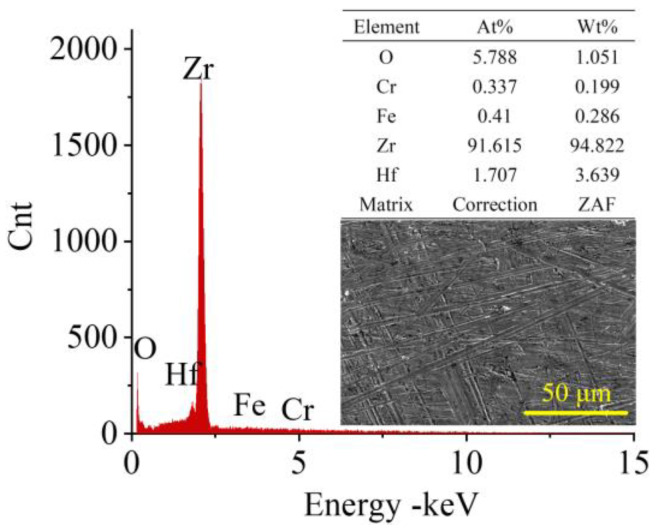
Chemical composition of the original Zr702 workpiece.

The experimental workpieces used in this study were Zr702 sheets measuring 25 × 25 × 0.5 mm. The chemical composition of Zr702 is presented in Figure 2.

### 2.2. Experimental Device

**Figure 3:**
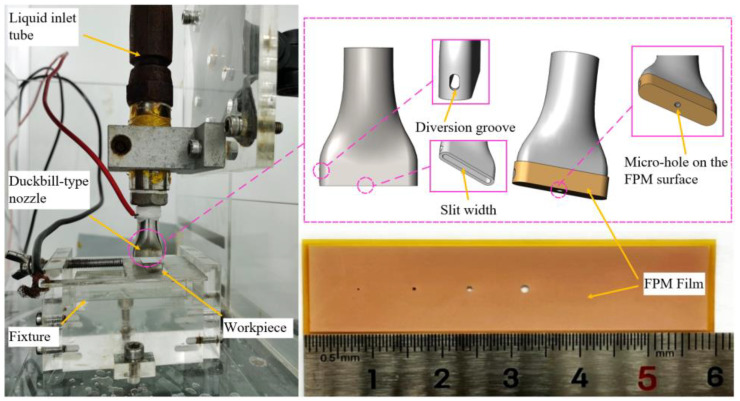
Schematic diagram of experimental device.

Figure 3 presents a schematic overview of the ECDW process, as well as a visual representation of the experimental setup employed in our study. The experimental apparatus consists of a three-coordinate micro-displacement platform. In this setup, a stainless-steel duckbill-type nozzle acts as the tool electrode, with a 1 mm slit width at the nozzle exit to facilitate efficient extrusion of the viscous PAM electrolyte. The nozzle surface is coated with an insulating film. The electrolyte is dispensed from the nozzle and directed onto the workpiece surface through an array of micro-holes in the insulation film. The insulation film, comprised of a flexible polyimide material (FPM), has a thickness of 200 μm. The FPM film is overlaid on the nozzle surface, and the electrolyte output is precisely regulated by a high-pressure pump, extruding through the micro-holes on the FPM surface. Through laser processing of the prepared FPM film, through-holes with different diameters are created as extrusion outlets. Additionally, various patterns can be etched onto the insulation membrane to meet the requirements of patterned microstructure direct writing processing. A diversion groove is located on one side of the nozzle to allow the excess PAM electrolyte to drain out, thus preventing the insulation membrane from being compressed. The workpiece is mounted on an insulated plate, while the nozzle moves in the negative *Z*-axis direction to make contact with the workpiece.

A patterned microstructure was created on the workpiece through electrochemical dissolution. During the machining process, the nozzle acted as the cathode and the workpiece as the anode. The PAM electrolyte was continuously dispensed onto the processing area via the nozzle.

The experimental device system was operated by an industrial computer developed by Mitsubishi Corporation (Tokyo, Japan). A SOYI-20050DM pulsed power supply (Soyi Power System Equipments, Shanghai, China) was utilized, and the fixture and electrolysis cell were constructed from acrylic material.

### 2.3. Experimental Design

In this study, four experiments were conducted. The first experiment focused on conducting polarization curve tests. Electrochemical corrosion polarization curves of Zr702 in different PAM electrolyte concentrations were examined using an electrochemical workstation (Zennium E). Their passivation and corrosion properties were compared. The viscosity of PAM at different concentrations was also measured in order to optimize the electrolyte concentration. The second experiment involved analyzing corrosion morphology and energy spectrum. By assessing Zr702 dissolution at a representative potential from the polarization curves, the electrochemical corrosion mechanism was elucidated. NaCl, a common electrolyte, was introduced as a control group in order to better study the electrolytic properties of PAM. The relationships between the current efficiency (η) and lateral corrosion coefficient (μ) were studied. The final experiment entailed a single-factor experiment on Zr702 sheet microgroove array machining. ECDW was utilized to assess the effects of electrolyte pressure, nozzle scan speed, PAM concentration, and pulse voltage on various shape accuracies (EF). Subsequently, experimental parameters were optimized to achieve patterned microgrooves with superior overall quality.

**Figure 4:**
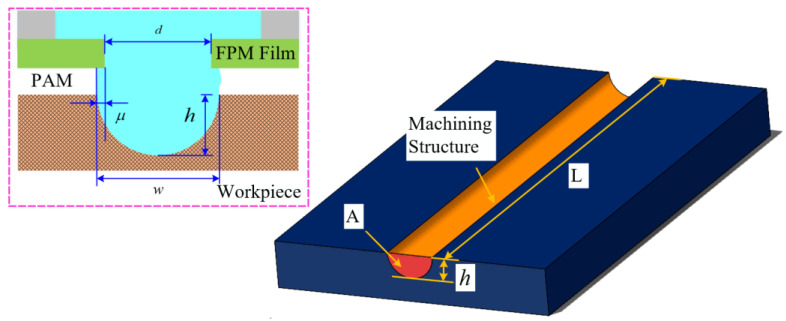
Distribution of sampling positions and characterization parameters.

A schematic diagram of the microstructure is presented in the left panel of Figure 4.

The width of the microstructure is $w_{i}$, the depth is $h_{i}$, and the diameter of the FPM aperture is d. In addition, μ is the lateral erosion coefficient. The accuracy of the width can be determined by calculating the average width (w) and average depth (h), as shown in Equations (8) and (9):

(8) $$w=\frac{\sum_{i=1}^{n}w_{i}}{n}$$

(9) $$h=\frac{\sum_{i=1}^{n}h_{i}}{n}$$

The shape accuracy is mainly indicated by the value of EF. According to Wu et al. [17], a higher value of EF corresponds to less lateral erosion of the microstructure, suggesting excellent localization.

(10) $$EF=\frac{h}{\mu }=\frac{2h}{w-d}$$

The material removal rate (MRR) is a critical parameter for describing machining efficiency, typically expressed as the amount of material electrochemically dissolved per unit time. It is calculated as the volume of material removed (V) during machining time (t). The MRR can be represented as

(11) V=L⋅A=v⋅t⋅A

(12) $$MRR=\frac{V}{t}=\frac{v\cdot t\cdot A}{t}=v\cdot A$$

where L represents the length of the machining, A denotes the cross-sectional area of the microstructure, and v is the scanning speed.

In practical machining, considering the influence of pulse duty cycle on machining results, the effective material removal rate within the effective time ($t_{on}$) is denoted as $MRR_{e}$. The theoretical material removal rate is denoted as $MRR_{t}$. It can be expressed as

(13) $$MRR_{e}=\frac{V}{t_{on}}=\frac{v\cdot t\cdot A}{t_{on}}=\frac{v\cdot A}{\varepsilon }$$

(14) $$MRR_{t}=\frac{V}{t}=\frac{\omega \cdot t\cdot I}{t}=\omega \cdot I$$

where ω represents the material’s volume electrochemical equivalent, and I denotes the average current. The relationship between the average current and the peak current ($I_{p}$) and duty ratio (ε) is given by

(15) $$I=I_{p}\cdot \varepsilon$$

Therefore, the $MRR_{t}$ can also be represented as

(16) $$MRR_{t}=\omega \cdot I_{p}\cdot \varepsilon$$

The current efficiency (η) in electrochemical machining can be expressed as

(17) $$\eta =\frac{MRR}{MRR_{t}}=\frac{v\cdot A}{\omega \cdot I_{p}\cdot \varepsilon }$$

The experimental parameters for the viscosity test are listed in Table 1. Table 2 presents the experimental parameters used in the polarization curve test, while Table 3 displays the single-factor experimental parameters. Each set of single-factor experiments was repeated three times, and the average results were calculated for further analysis.

**Figure 5:**
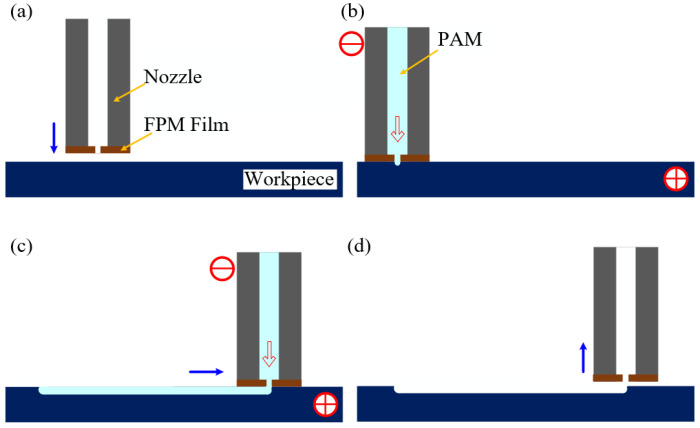
ECDW process: (**a**) the metal nozzle with FPM; (**b**) PAM flows towards the workpiece surface under the guidance of a template; (**c**) the relative motion between the nozzle and the workpiece; (**d**) nozzle leaves the workpiece.

The processing procedure is depicted in Figure 5. Initially, as illustrated in Figure 5a, the metal nozzle with FPM descends vertically along the *Z*-axis to approach the workpiece. Upon achieving close proximity between the nozzle and the workpiece, PAM flows towards the workpiece surface under the guidance of a template (Figure 5b). The workpiece is electrically connected to the positive terminal and the nozzle to the negative terminal of the power supply. During processing, electrolytic corrosion progresses in the depth direction. Concurrently, the relative motion between the nozzle and the workpiece enables scanning from point to line, facilitating the formation of microgrooves, as depicted in Figure 5c. Upon completion of the machining process, the power supply is deactivated, the liquid flow ceases, and the nozzle ascends along the *Z*-axis, retracting from the workpiece surface, thereby concluding the microgroove machining process, as shown in Figure 5d. Throughout the operation, adjustment of the nozzle trajectory allows for the attainment of diverse patterned microstructures.

The electrochemical performance test used a three-electrode system, with a platinum sheet (20 mm × 20 mm × 0.1 mm) as the reference electrode. The sectional profiles of the microstructures were evaluated using a confocal laser scanning microscope (LEXT OLS4000, Olympus, Tokyo, Japan), with measurements taken at five points for each microstructure. The pulse current during machining was monitored with a current sensor operating at a sampling frequency of 100 kHz. Scanning electron microscope (SEM) images of the microhole arrays were obtained using an S-3400N model from Hitachi, Japan. The viscosity of the PAM electrolyte at various concentrations was determined using a rotational rheometer (HAAKE Mars60, Thermo Fisher Scientific, Waltham, MA, USA).

## 3. Results and Discussion

### 3.1. Electrochemical Properties of Zr702 with PAM

**Figure 6:**
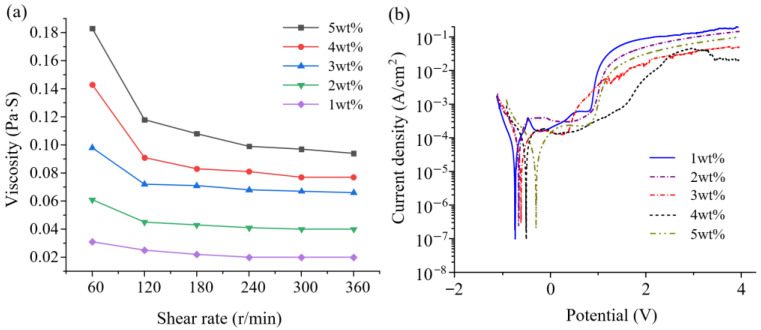
Electrochemical dissolution polarization curves for Zr702 in PAM electrolyte: (**a**) viscosity of PAM electrolyte; (**b**) different concentrations.

Non-Newtonian fluids do not follow Newton’s law of viscosity, showing a nonlinear relationship between shear stress and shear strain rate. PAM, a common non-Newtonian fluid, exhibits unique physical properties. In ECM, the viscosity of PAM plays a crucial role in determining the flow field distribution and ensuring the confinement of air bubbles. Additionally, the electrolyte viscosity dictates the extrusion pressure through the FPM film. As illustrated in Figure 6a, the viscosity of the PAM electrolyte is significantly higher than that of traditional electrolytes. Moreover, the viscosity decreases with increasing shear rate, demonstrating shear thinning behavior. As the PAM concentration increases, its viscosity rises markedly, necessitating higher pressures for extrusion through the FPM film when the viscosity becomes excessively high.

The rate of electrolysis is positively correlated with the concentration of polar groups (–CONH_2_). Additionally, the formation of Zr^4+^ ions increases as the current density increases, leading to higher levels of metal complexes being generated. The polarization curve had a potential scanning range of −2 V to 4 V, with a scanning speed of 50 mV/s and a voltage amplitude of 10 mV. A preferred concentration range of 1 wt%–5 wt% of PAM electrolyte (with a 1% interval for each concentration) was chosen for further optimization. As shown in Figure 6b, the current density (lgi) values during the passivation interval with various PAM electrolyte concentrations (8 million molecular weight) are lower. The passivation threshold potential exhibits minimal changes with changing concentrations. The polarization curve displays a distinct passivation region, suggesting an extended duration for the penetration of the oxide layer on the workpiece within the PAM solution, resulting in a relatively lower corrosion rate within this region. Beyond 1 V, a sharp increase in current density is observed, followed by a plateau as the potential continues to increase. This phenomenon is attributed to overpassivation; when overpassivation occurs, the oxide film breaks down, leading to the removal of the anode material.

In general, the lgi within the passivation range shows a gradual increase. The lowest recorded lgi value within the passivation range was 1.318 × 10^−4^ A/cm^2^ at a concentration of 4 wt%. The highest self-corrosion potential was −0.124 V at 5 wt%. Considering both electrochemical corrosion mechanisms and processing efficiency, a concentration of 5 wt% PAM electrolyte was selected as the electrolyte concentration for subsequent processing.

Table 4 outlines some of the key parameters to be used in the upcoming PAM studies, which will be conducted in four stages.

### 3.2. Analysis of Zr702 Electrochemical Dissolution Characteristics

**Figure 7:**
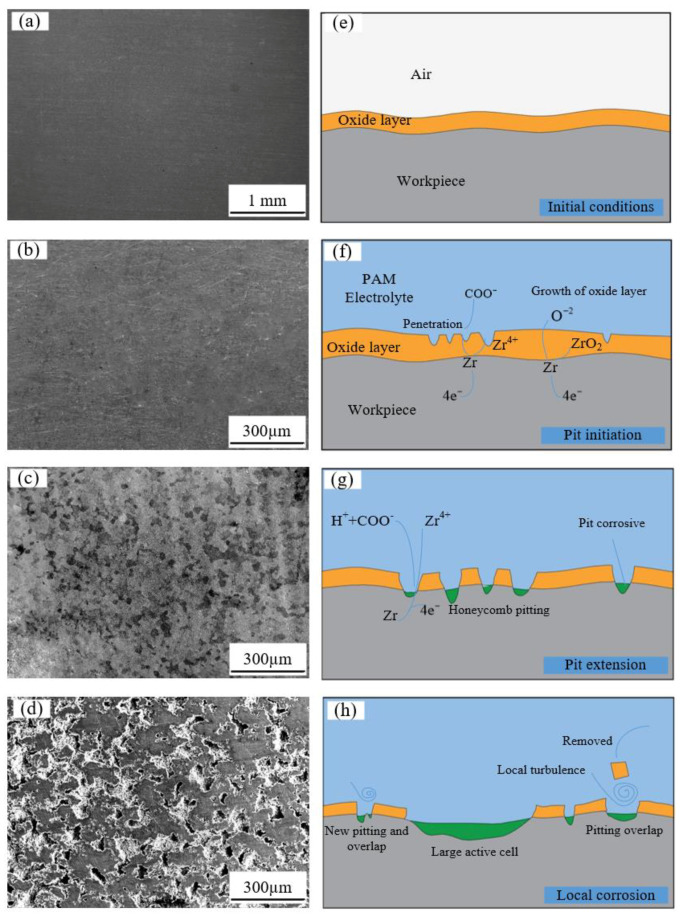
Surface corrosion morphologies of Zr702 in PAM at 5 wt% concentration. (**a**) −0.124 V; (**b**) −0.085 V; (**c**) 0.241 V; (**d**) 2.000 V; (**e**–**h**) are schematic diagrams of the material removal mechanisms for (**a**–**d**).

Figure 7 displays the surface corrosion morphologies of Zr702 at various potentials. In stage 0, as shown in Figure 7a, the original Zr702 surface exhibits almost no electrochemical corrosion. Subsequent activation and stable passivation potentials, as depicted in Figure 7b,c, also do not show significant corrosion on the Zr702 surfaces. Although theoretically, the workpiece should corrode when an activation potential is applied, no corrosion was observed, possibly due to the presence of a surface oxide film. Figure 7d indicates the beginning of dissolution on the Zr702 sample surface at the superpassivation potential, with pitting as the main form of corrosion. As the potential increased to 2.0 V, the pits expanded and merged, forming larger corrosion pits. The surface corrosion of the Zr702 samples at activation and stable passivation potentials appeared uneven.

The key to achieving electrochemical machining of zirconium metal lies in the effective removal of the passivation film. As the potential increases, the passivation film gradually peels away, allowing the zirconium substrate to undergo oxidative dissolution through electrochemical reactions, thereby enabling the machining of zirconium material. As illustrated in Figure 7e–h, the dissolution process of Zr702 can be broadly divided into four stages.

Figure 7e depicts the original, unprocessed Zr702 workpiece, with a thin oxide layer forming on the surface due to air oxidation. At the beginning of electrochemical machining, as shown in Figure 7f, the applied voltage causes the electrolyte to ionize, and PAM begins to act by gradually replacing the oxygen elements in the oxide film. This leads to localized depassivation of the Zr702 oxide layer at defect sites. Upon contact with elemental zirconium, ${\mathrm{COO}}^{-}$ ions oxidize it to Zr^4+^ under electrochemical action. With an increase in applied voltage, the activity of ${\mathrm{COO}}^{-}$ ions further intensifies, leading to deeper erosion into the oxide layer. This causes the oxide layer to breach and rupture locally, exposing the zirconium substrate directly to the electrolyte. These exposed areas exhibit higher current density, resulting in localized dissolution of the zirconium metal and pitting corrosion.

As the reaction progresses, ${\mathrm{COO}}^{-}$ ions continue to react with Zr^4+^ ions, forming higher-valence zirconium compounds and further eroding the oxide layer. The localized pitting regions expand, and under the influence of electrolyte flow, portions of the oxide layer detach, leading to an enlargement of the corrosion area as depicted in Figure 7g. Through continued machining, the oxide layer is gradually and completely removed, allowing more Zr^4+^ ions to enter the electrolyte, resulting in the electrochemical machining effect. Eventually, the Zr702 metal reaches a stable dissolution state, as shown in Figure 7h.

**Figure 8:**
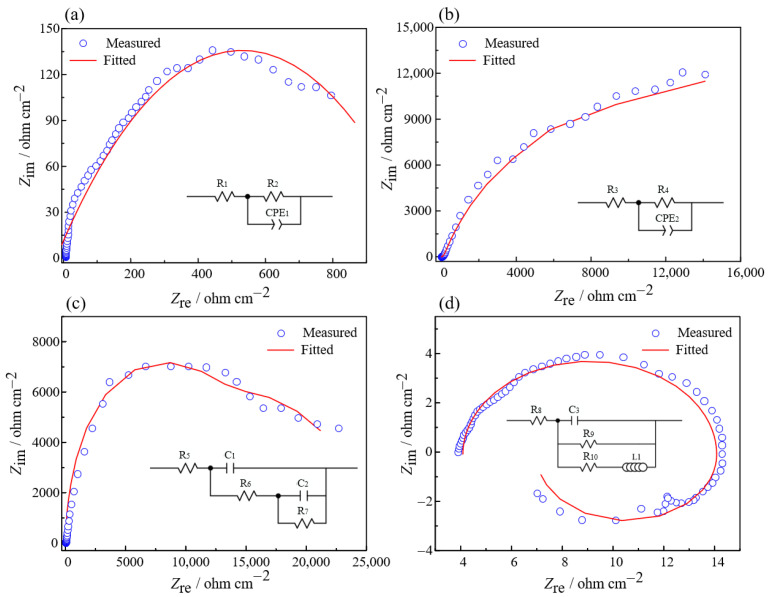
EIS with PAM electrolyte: (**a**) Stage 0; (**b**) Stage 1; (**c**) Stage 2; (**d**) Stage 3.

Based on the results of electrochemical impedance spectroscopy (EIS), the high-frequency region of the Nyquist diagram at the self-corrosion potential displays a capacitive arc, as shown in Figure 8a. At this point, the resistance value R_1_ of the solution is only 2.86 × 10^−8^ Ω·cm^−2^, indicating that the passive film on the surface is primarily composed of Zr material before the electrochemical reaction, while the resistance value R_2_ is 1183.01 Ω·cm^−2^. There is a slight dispersion phenomenon in the low-frequency region, suggesting that the Zr element is actively dissolving, with some of the Zr^4+^ ions, generated by oxidation, diffusing into the electrolyte.

In Stage 1, the high-frequency region of the Nyquist diagram presents a semicircle that consists of a large capacitive reactance arc, as shown in Figure 8b. The resistance value R_3_ of the solution increased to 188.01 Ω·cm^−2^, while the resistance value R_4_ for the passivation film increased dramatically to 31,847.56 Ω·cm^−2^. During this period, there was a notable increase in oxygen content compared to the self-corrosion stage, indicating a corresponding increase in the passivation film’s thickness. At this stage, the Zr base metal is not actively dissolving, with the oxide film forming at a rate greater than its dissolution rate.

Moving on to Stage 2, as shown in Figure 8c, the Nyquist diagram displays a semicircular arc in the high-frequency region attributable to the electric double-layer structure, while another semicircular arc appears in the low-frequency region due to the ZrO_2_ insulating passivation film. The resistance of the passivation film at this point drops to 15,631.44 Ω·cm^−2^ compared to Stage 1, signifying a decrease in oxygen content. These observations suggest a reduction in the thickness of the passivation film, resulting in a dynamic equilibrium between its formation and dissolution.

The high-frequency region of the Nyquist diagram for Stage 3 consists of a capacitive arc, as shown in Figure 8d. The resistance of the passivation film, which is only 12.96 Ω·cm^−2^, is significantly reduced when compared to Stage 2. This reduction in resistance is accompanied by a drastic decrease in oxygen content, suggesting a substantial reduction in the thickness of the ZrO_2_ passivation film. Inductive arcs emerge in the low-frequency region as the corrosive ${\mathrm{COO}}^{-}$ is adsorbed on the Zr material’s surface. This process leads to the gradual breakdown of the ZrO_2_ layer, allowing the corrosive agents to infiltrate the Zr base metal and initiating the formation of pits on the Zr material’s surface. Following Stage 3, the corrosion rate of the Zr increases sharply.

### 3.3. Experimental Machining Results

**Figure 9:**
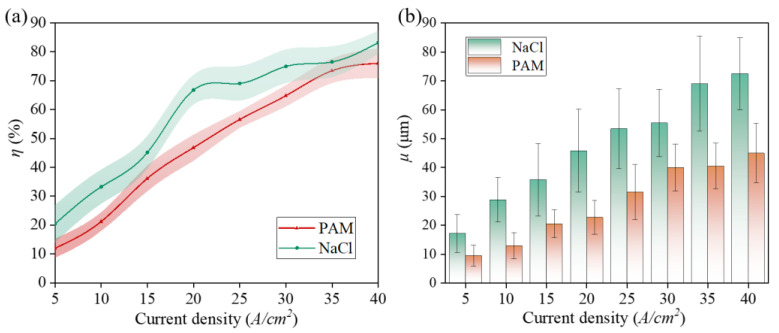
Zr702 in PAM and NaCl electrolytes (5 wt%): (**a**) η and (**b**) μ.

To further investigate the electrolytic properties of PAM, a common electrolyte, NaCl, was introduced as a control group. Figure 9a illustrates the relationship between current efficiency (η) and current density for Zr702 in 5 wt% NaCl and PAM electrolytes. η is observed to increase as current density rises. For any given current density, the η value for NaCl is consistently higher than that for PAM. In the current density range of 5–15 A/cm^2^, the η value for the PAM electrolyte increases rapidly. Between 30–40 A/cm^2^, η continues to rise with increasing current density, although at a slower pace. At a current density of 40 A/cm^2^, the η value for the NaCl electrolyte reaches approximately 84.5%, compared to 77.4% for the PAM electrolyte. The 7.1% difference in the current efficiencies suggests that the electrochemical machining performance of the PAM electrolyte is fairly comparable to that of the NaCl electrolyte.

As shown in Figure 9b, the lateral etching coefficients (μ) of the two different electrolytes differ significantly. NaCl, a traditional electrolyte, demonstrates a higher lateral etching coefficient due to its physical properties during processing, resulting in lower machining accuracy. In contrast, PAM, a non-Newtonian fluid, possesses higher viscosity and poorer flowability when applied to the workpiece. This characteristic allows for more specific action of the current density in targeted areas, thereby improving localization and machining accuracy.

**Figure 10:**
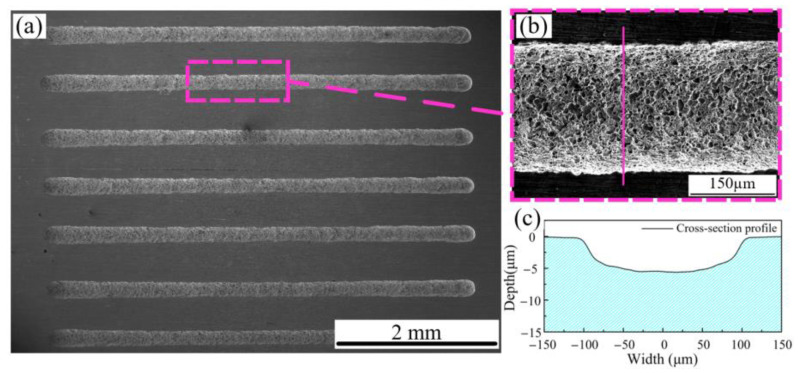
Machining experiments with PAM electrolyte: (**a**) microgroove machining; (**b**) magnified view; (**c**) cross-sectional view.

As shown in Figure 10, machining experiments were conducted with the following parameters: FPM aperture of 150 μm, PAM concentration of 5%, electrolyte pressure of 30 kPa, nozzle movement speed of 5 μm/s, and a voltage of 10 V. Other parameters are listed in Table 4. Seven experiments were performed. The machining results indicate that the microgroove structure is relatively complete with good surface quality. μ is approximately 20 μm, demonstrating good localization. However, the depth only reaches around 6–8 μm, reflecting the electrochemical machining capability of PAM. Figure 10b provides a magnified view of the microgroove, while Figure 10c shows a cross-sectional image.

**Figure 11:**
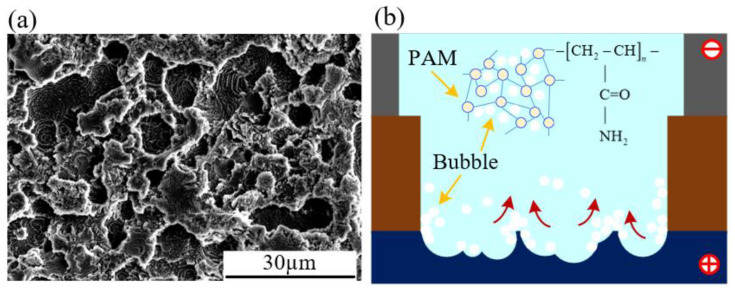
(**a**) Microgroove bottom; (**b**) bubble attachment diagram.

As shown in Figure 11a, the magnified view of the microgroove bottom reveals a dense distribution of small pit-like structures. This phenomenon is likely attributable to the non-Newtonian fluid properties of PAM, which hinder the prompt escape of bubbles generated during machining. Consequently, these bubbles accumulate on the workpiece surface, as shown in Figure 11b. This accumulation can reduce the current density in localized areas, resulting in a lower material removal rate and the formation of pit-like structures. Additionally, the high viscosity of PAM facilitates the retention of electrolysis byproducts in the machining area, further affecting the distribution of the current density.

**Figure 12:**
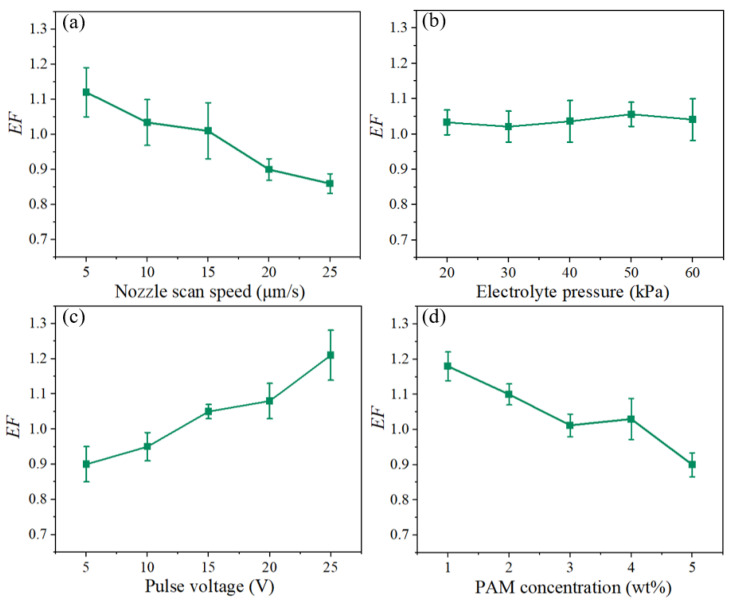
Variation trends of EF with (**a**) nozzle scan speed (30 kPa, 5 V, 5 wt%); (**b**) electrolyte pressure (5 μm/s, 5 V, 5 wt%); (**c**) pulse voltage (5 μm/s, 30 kPa, 5 wt%); (**d**) PAM concentration (5 μm/s, 30 kPa, 5 V).

A single-factor experiment for EF was also conducted. The experimental parameters are listed in Table 4, and the results are shown in Figure 12. As illustrated in Figure 12a,d, the EF of the microgroove structure on the workpiece surface gradually decreases with an increase in the nozzle movement speed. This is because the interaction time per unit decreases as the nozzle movement speed increases, causing both w and h to decrease accordingly. Viscosity also increases with an increase in concentration. Under the same electrolyte pressure, diffusion of the electrolyte on the workpiece surface decreases, leading to higher localization. Consequently, EF decreases. As shown in Figure 12b, when the electrolyte pressure increases, there is no significant change in EF. This is because the high viscosity of the non-Newtonian fluid electrolyte makes it difficult for machining bubbles and byproducts to leave the machining surface under the same processing parameters, thus affecting material removal. In ECDW, electrolyte renewal at the machining site is achieved by moving the nozzle position, which means that the material removal rate is less affected by pressure. Figure 12c shows that with an increase in the applied voltage, the EF value increases. This is because the increase in voltage enhances the efficiency of electrochemical machining, enabling more material to be removed. Both depth and width increase accordingly, but due to the attachment of the byproducts and bubbles, material in the depth direction is removed more easily, causing the EF to increase.

**Figure 13:**
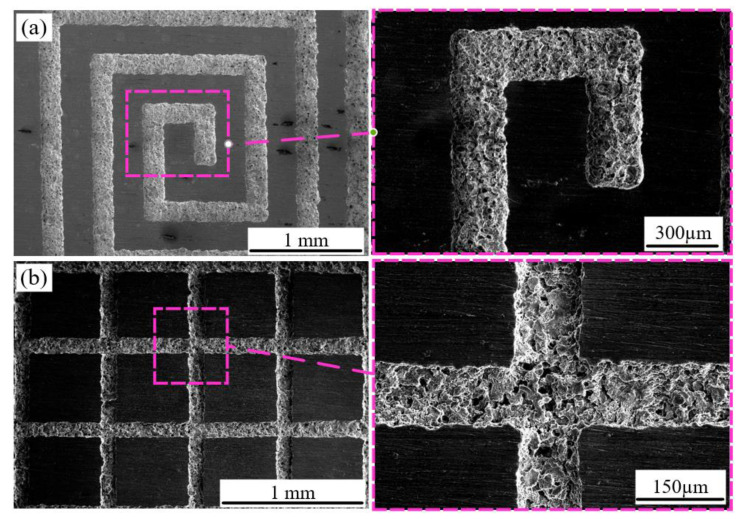
Morphology of microstructure processed using ECDW: (**a**) FPM aperture of 150 μm; (**b**) FPM aperture of 80 μm.

Based on the results of the single-factor experiment, the ECDW patterned microstructure was fabricated with optimal experimental parameters (25 μm/s, 30 kPa, 5 V, 5 wt%). As shown in Figure 13a, microgroove patterned machining was performed with good machining quality, indicating that ECDW can be effectively conducted using PAM. The EF value was found to be 0.951. Additionally, to enhance machining efficiency, a series of micro-holes were fabricated on FPM. The arrayed microgroove machining results were successfully achieved by extruding the PAM electrolyte, as shown in Figure 13b.

## 4. Conclusions

This paper proposes a maskless ECDW technique using a PAM polymer electrolyte. The method takes advantage of the unique non-Newtonian properties of PAM to confine the electrolyte, thereby addressing the stray corrosion issue in traditional ECM and improving machining localization. The polarization curves of Zr702 in PAM electrolyte were examined, along with the viscosity variation characteristics of PAM and EIS, to elucidate the electrochemical properties of Zr702 and demonstrate its machinability under the influence of PAM. Single-factor experiments were conducted on parameters such as nozzle scanning speed, electrolyte pressure, voltage, and PAM concentration. The results indicated that the EF value in the PAM electrolyte is smaller than that in the traditional NaCl electrolyte. The EF of the microstructures is minimally affected by electrolyte pressure, increases with voltage, and decreases with nozzle movement speed and PAM concentration. Based on these experiments, patterned microstructure fabrication experiments were conducted, resulting in patterned groove structures and array groove structures. The findings demonstrate that PAM’s properties can be utilized for the electrochemical machining of Zr702, with its non-Newtonian fluid characteristics contributing to improved machining accuracy and localization.

## Figures and Tables

**Table 1:** Experimental parameters for viscosity test.

Parameter	Value
PAM (wt%)	1, 2, 3, 4, 5
Shear rate (rpm)	120, 180, 240, 300, 360
Time (s)	30
Pressure (kPa)	101.325
Temperature (°C)	25

**Table 2:** Experimental parameters for polarization curve test.

Parameter	Value
PAM concentration (wt%)	1, 2, 3, 4, 5
Measuring potential (V)	−2 to 4
Scan rate (mV/s)	1
Temperature (°C)	25

**Table 3:** Single-factor experimental parameters.

Parameter	Value
Nozzle scan speed (μm/s)	5, 10, 15, 20, 25
Electrolyte pressure (kPa)	20, 30, 40, 50, 60
Pulse voltage (V)	5, 10, 15, 20, 25
PAM concentration (wt%)	1, 2, 3, 4, 5
pulse frequency (Hz)	1000
FPM aperture (μm)	150
Duty ratio (%)	50

**Table 4:** Polarization curve parameters for Zr702 in PAM at 5 wt%.

Category	Original Zr702	Self-Corrosion Potential	Activation Potential	Stable Passivation Potential	Threshold Passivation Potential
Stage	0	/	1	2	3
PAM	/	−0.124 V	−0.085 V	0.241 V	0.737 V

## Data Availability

All data used in this study have been properly cited within the article.
